# Physical inactivity in healthy, obese, and diabetic adults in Germany: An analysis of related socio-demographic variables

**DOI:** 10.1371/journal.pone.0246634

**Published:** 2021-02-09

**Authors:** Stephanie Linder, Karim Abu-Omar, Wolfgang Geidl, Sven Messing, Mustafa Sarshar, Anne K. Reimers, Heiko Ziemainz

**Affiliations:** 1 Division of Physical Activity and Public Health, Department of Sport Science and Sport, Friedrich-Alexander University Erlangen-Nuremberg, Erlangen, Bavaria, Germany; 2 Division of Exercise and Health, Department of Sport Science and Sport, Friedrich-Alexander University Erlangen-Nuremberg, Erlangen, Bavaria, Germany; 3 Division of Health and Physical Activity, Department of Sport Science, Otto-von-Guericke University, Magdeburg, Germany; Dasman Diabetes Institute, KUWAIT

## Abstract

**Background:**

Adults with diabetes or obesity are more likely to be physically inactive than healthy adults. Physical activity is essential in the management of both diseases, necessitating targeted interventions in these groups. This study analysed physical inactivity (defined as not taking part in leisure-time physical activity) in over 100,000 adults in Germany considering their body mass index and the presence of diabetes. Furthermore, the relationship between specific socio-demographic factors with physical inactivity was investigated, particularly focussing diabetic and obese people, to refine the identification of risk-groups for targeted interventions on physical activity promotion.

**Methods:**

Data from 13 population-based health surveys conducted in Germany from 1997 to 2018 were used. The relevant variables extracted from these datasets were merged and employed in the analyses. We included data from 129,886 individuals in the BMI analyses and 58,311 individuals in the diabetes analyses. Logistic regression analyses were performed to identify the importance of six socio-demographic variables (age, sex/gender, education, income, employment, and migration) for the risk of physical inactivity.

**Results:**

Obese and diabetic people reported a higher prevalence of physical inactivity than those who were not affected. Logistic regression analyses revealed advanced age, low education level, and low household income as risk factors for physical inactivity in all groups. A two-sided migration background and unemployment also indicated a higher probability of physical inactivity.

**Conclusion:**

Similar socio-demographic barriers appear to be important determinants of physical inactivity, regardless of BMI status or the presence of diabetes. However, physical activity promoting interventions in obese and diabetic adults should consider the specific disease-related characteristics of these groups. A special need for target group specific physical activity programmes in adults from ethnic minorities or of advanced age was further identified.

## Introduction

Diabetes and obesity are critical challenges to public health [1]. In Germany, nearly five million adults have diabetes [2, 3]. A similar pattern is present for obesity (body mass index (BMI) ≥ 30 kg/m^2^), with almost a quarter of the German population affected [4]. In addition, being obese [5] is a crucial risk factor for the development of type 2 diabetes [3, 6].

Studies have highlighted physical inactivity as a major modifiable lifestyle factor in the prevention and management of both conditions [7, 8] since increasing physical activity (PA) precipitates significant health and weight loss benefits [9]. Nevertheless, adults with diabetes or obesity continue to be particularly inactive and are more likely to fail to meet PA recommendations than healthy individuals [10, 11]. Public health professionals have therefore identified the need for programs to promote PA in these groups [12]. Many studies have attempted to better understand the different propensities of physical inactivity, consequently, they have identified variables that influence people’s PA behaviour. In addition to age and sex/gender [13], socio-demographic factors, particularly income, education, employment status, and migration background, have been repeatedly proven to influence PA [14–18]. An analysis of factors that influence PA behaviour is essential to develop appropriate PA interventions [19, 20]. However, due to the generally small number of study participants, socio-demographic factors associated with physical inactivity have not yet been extensively examined among people with obesity or diabetes. Mapping and characterising such factors and potential social gradients for different sub-groups of obese people could refine the identification of target groups to enhance the tailoring of interventions designed to promote PA. Additionally, such an investigation is necessary to assess whether socio-demographic factors for physical inactivity may differ across vulnerable groups.

While there is growing evidence, that in particular PA in the context of leisure time has promoting health benefits [21, 22], accurate data on leisure-time PA among obese and diabetic individuals in Germany remains scarce. Therefore, the present study investigates physical inactivity defined as not taking part in any leisure-time PA among individuals with normal weight, overweight or obesity and with or without a diagnosis of diabetes using a large sample of adults in Germany. To uncover a detailed picture of physically inactive individuals, we further stratified results by age, educational level, and household income. Since the evidence indicates several socio-demographic factors which influence physical inactivity, a further goal was to analyse the association between different socio-demographic variables for physical inactivity stratified by BMI and the presence or absence of diabetes. Our analysis intends to provide new insights into the propensity of physical inactivity at leisure time and its socio-demographic predictors among obese and diabetic individuals to support the future adoption and tailoring of PA promotion programs for these risk groups.

## Method

### Participants and data collection

This analysis was based on pooled data from 13 population-based national health surveys (conducted between 1997 and 2018) involving 143,709 participants. Seven surveys were part of a nationwide Health Monitoring System administered by the Robert Koch-Institute [23–29] four surveys were conducted by the Leibniz Institute for the Social Sciences [30–33] and one each by the Max Planck Institute for Social Law and Social Policy [34–36] and the GFK Nürnberg [37]. All surveys were self-reported and comprised several major sections related to information about general socio-demographic and socio-economic characteristics, health status, and PA behaviour. Since we conducted a secondary data analysis of the given data sets, ethical approval is not applicable. An overview of the included surveys is presented in Table 1.

**Table 1:** Data sets included in the analysis.

Year(s) data were collected	Survey	Institution collecting the data	Age range of respondents	Sample size
1997–1999	BGS 98	Robert Koch Institute	18–79	7,124
1998	Allbus	Leibniz Institute for the Social Sciences	18+	3,234
2003	GSTel03	Robert Koch Institute	18+	8,318
2004	Allbus	Leibniz Institute for the Social Sciences	18+	2,946
2006	NBS	GFK Nürnberg	10+	10,554
2008–2011	DEGS1	Robert Koch Institute	18–79	7,987
2009	GEDA 2009	Robert Koch Institute	18+	21,262
2010	GEDA 2010	Robert Koch Institute	18+	22,050
2012	GEDA 2012	Robert Koch Institute	18+	19,294
2013	SHARE-WAVE 5	Max Planck Institute for Social Law and Social Policy	50+	5,752
2013–2018	GESIS Panel	Leibniz Institute for the Social Sciences	18+	7,599
2014	Allbus	Leibniz Institute for the Social Sciences	18+	3,471
2014	GEDA 2014	Robert Koch Institute	18+	24,016

### Study design

Two sub-samples were formed by stratifying the total population based on BMI and diabetes. The ‘BMI sample’ included data from adults aged 18 years and older. The final sample consisted of 129,886 individuals. The ‘diabetes sample’ exclusively included people aged 50 years and over because in more than 50% of the cases, diabetes is diagnosed at or after the age of 50 years [38]. The final sample consisted of 58,311 individuals. Individuals with BMI < 18.5 kg/m^2^ were excluded from the analysis.

### Specificities of the sub-samples

#### BMI sample

Based on the self-reported height and weight data from the surveys, BMI was calculated as weight in kilograms divided by the square of height in metres. The participants were divided into five categories based on the classification of the World Health Organisation [5]: normal weight (18.5 to <25 kg/m^2^), overweight (25 to <30 kg/m^2^), obesity grade I (30 to <35 kg/m^2^), obesity grade II (35 to <40 kg/m^2^) and obesity grade III (≥ 40 kg/m^2^).

#### Diabetes sample

Different questions were used to identify diabetes in the surveys: participants were asked if they suffer from diabetes, had ever received a medical diagnosis, or received a diagnosis within the last 12 months. Participants who answered ‘No’ to these questions were included in the non-diabetic group, and those who answered ‘Yes’ were included in the diabetic group.

### Measures

#### Physical inactivity

PA was assessed related to the duration of leisure-time PA per week within the last three months (e.g., GEDA 2009) or generally (e.g., GESIS Panel, Allbus). Regarding the information on weekly duration, the responses were grouped in hours as follows: no PA, ≤2 h, 2–4 h, and >4 h. Most datasets included questions on leisure-time PA only (BGS 1998, GEDA 2009, 2010, 2012, SHARE 5, DEGS1). Adults were identified as being physically inactive and thus assigned to the group ‘no PA’ if they reported to never engage in leisure-time physical activities (BGS 1998, NBS 2006). Respondents to SHARE 5 were coded as being physically inactive if they reported almost never or never engaging in leisure-time PA. Respondents to GEDA 2009, GEDA 2010, GEDA 2012 and DEGS1 were assigned to the group ‘no PA’ if they engaged in no leisure-time PA in the past week. Adults who indicated that they were physically active were allocated to the groups ‘≤2 h’ to ‘>4 h’ according to their stated duration of leisure-time PA.

#### Socio-demographic factors

Sine we pooled secondary data sets the analysis of socio-demographic variables was restricted to those that are routinely collected as part of behavioural risk factor surveillance. Participants answered basic questions about their socio-demographic background. Six socio-demographic variables were included in the analysis. For each variable separate categories were formed: (1) sex/gender (male, female); (2) age (for BMI sample: 18 -< 30 years, 30 -< 60 years, 60 -< 80 years, and 80+ years; for diabetes sample: 50 -< 60 years, 60 -< 70 years, 70 -< 80 years, and 80+ years); (3) education level (low (9–10 years schooling), medium (10–11 years schooling), and high (12–13 years schooling)); (4) household income (bottom 20%, 20–40%, 40–60%, 60–80%, and top 20%); (5) migration background (none, one-sided, and two-sided) and (6) employment (unemployed, employed).

### Statistical analysis

All analyses were conducted separately for the BMI sample and the diabetes sample. The results of the analysis of the diabetes sample are presented in the appendix only.

Descriptive statistics (frequencies) were used to determine PA levels of all groups and to illustrate the distribution of physical inactivity (corresponds to the ‘no PA’ group) for the variables age, education level and household income. Binary logistic regression analyses were conducted to investigate the associations between the six socio-demographic variables with physical inactivity as being the dependent variable. The odds ratio (OR) was estimated for each BMI subgroup separately. This allows to investigate the socio-demographic correlates of not taking part in leisure-time PA for the different BMI subgroups. The significance level of all tests was based on *p* < .05. All analyses were performed using the IBM SPSS statistical software package, version 21.

## Results

### Sample characteristics

Table 2 presents the detailed characteristics of the whole sample. In all, 129,886 adults (44.8% male) were included; 47% of them were normal weight, 36.4% were overweight, and 16.6% were obese (grade I: 12.2%, grade II: 3.1%, grade III: 1.3%). Most adults were in the age group of 30–59 years (55.3%), had no migration background (86.8%), and were employed (79.6%).

**Table 2:** Sociodemographic characteristics by BMI subgroups in n (% cumulated).

	BMI subgroups (in kg/m^2^) (n = 129,886)				
Variables	18,5 -< 25 (n = 61,029)	25 -< 30 (n = 47,253)	30 -< 35 (n = 15,864)	35 -< 40 (n = 4,045)	≥ 40 (n = 1,695)
**Sex/Gender** (%Male)	23,479 (38.5)	26,930 (57.0)	8,057 (50.8)	1,681 (41.6)	612 (36.1)
**Employment** (% unemployed)	6,512 (16.1)	5,476 (18.0)	2,122 (20.9)	597 (22.9)	277 (24.1)
**Age** (in years)					
18 -< 30	14,166 (23.4)	4,260 (9.0)	1,010 (6.4)	237 (5.9)	138 (8.2)
30 -< 60	32,892 (54.2)	25,472 (54.0)	8,378 (52.9)	2,251 (55.7)	1,008 (59.7)
60 -< 80	11,950 (19.7)	15,885 (33.7)	5,975 (37.7)	1,468 (36.3)	499 (29.5)
80+	1,630 (2.7)	1,571 (3.3)	478 (3.0)	83 (2.1)	44 (2.6)
**Education level**					
Low	14,194 (24.2)	17,538 (38.6)	7,373 (48.4)	1,994 (51.3)	859 (52.2)
Middle	16,539 (28.2)	11,758 (25.9)	3,764 (24.7)	971 (25.0)	409 (24.8)
High	27,854 (47.5)	16,138 (35.5)	4,103 (26.9)	924 (23.8)	379 (23.0)
**Household income**					
Bottom 20%	10,687 (18.6)	7,252 (16.5)	2,997 (20.5)	918 (24.7)	481 (30.6)
20–40%	11,201 (19.4)	9,015 (20.5)	3,365 (23.1)	898 (24.2)	359 (22.8)
40–60%	12,527 (21.8)	10,272 (23.4)	3,392 (23.3)	825 (22.2)	305 (19.4)
60–80%	10,673 (18.5)	8,375 (19.1)	2,547 (17.5)	582 (15.7)	227 (14.4)
Top 20%	12,506 (21.7)	9,021 (20.5)	2,284 (15.7)	495 (13.3)	201 (12.8)
**Migration background**					
Non	28,025 (86.0)	20,907 (87.6)	6,514 (86.6)	1,626 (86.6)	670 (87.1)
One-sided	1,445 (4.4)	977 (4.1)	344 (4.6)	90 (4.8)	41 (5.3)
Two-sided	3,108 (9.5)	1,989 (8.3)	660 (8.8)	161 (8.6)	58 (7.5)
**Diabetes** (% diabetic)	1,431 (2.6)	3,061 (7.2)	2,074 (14.5)	867 (23.7)	407 (27.0)

### PA levels

**Fig 1:**
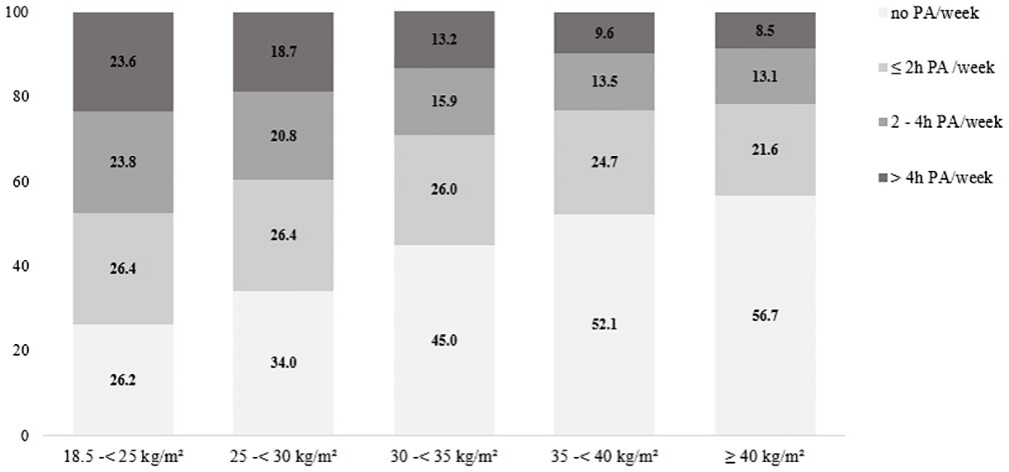
Percentage distribution of PA categories (frequencies) for the BMI sample.

The prevalence of physical inactivity was lowest among normal weight individuals (26.2%), followed by overweight individuals (34%) and was highest among people with obesity grade III (56.7%). Only small group differences were found for ‘≤2 h’, with percentages ranging between 21.6% (obesity grade III) and 26.4% (normal weight) and ‘2–4 h’, with percentages ranging between 15.9% (obesity grade I) and 23.8% (normal weight). Furthermore, 23.6% of the normal-weight individuals engaged in PA for more than four hours per week; lower percentages were found for overweight people (18.7%) and the lowest for individuals with obesity grade II (9.6%) and III (8.5%). An overview of the percentages of all groups is presented in Fig 1.

### Socio-demographic characteristics among inactive individuals by age, education and household income

**Fig 2:**
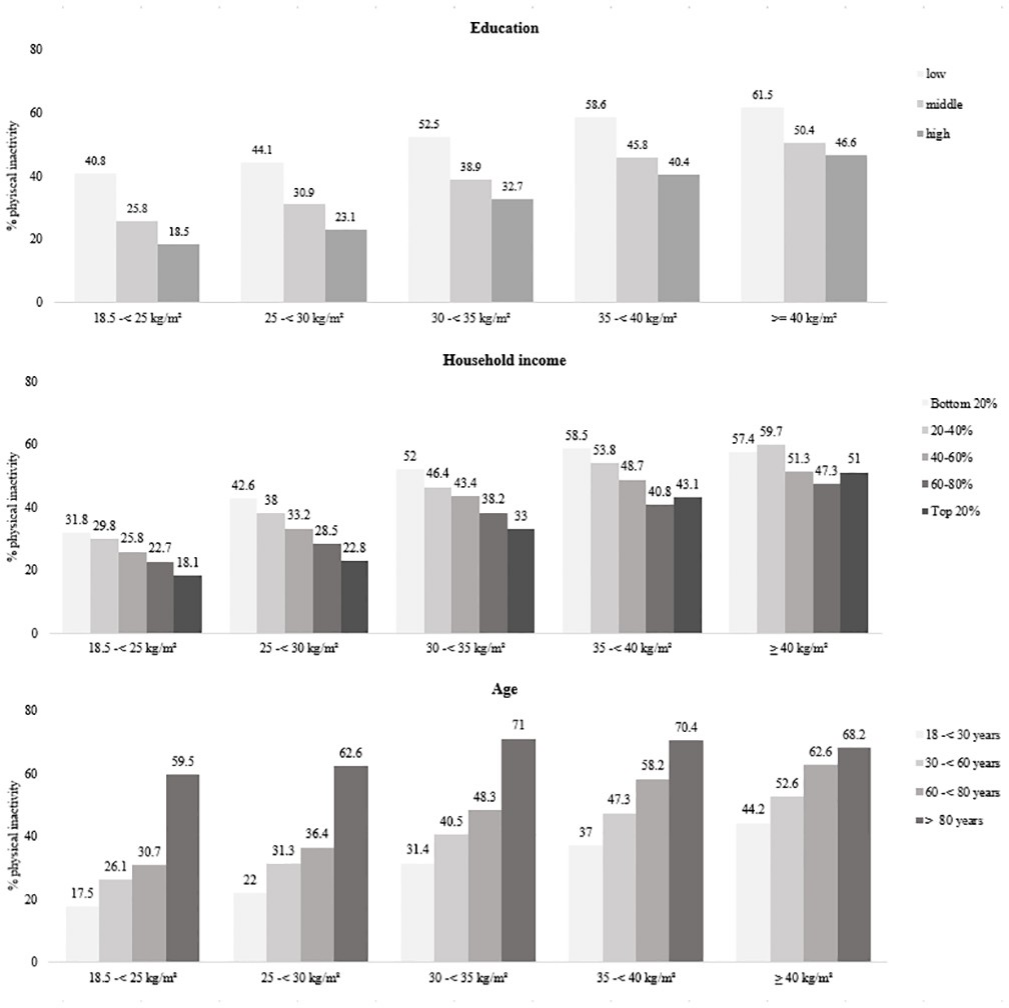
Physical inactivity (in %) for education, household income and age across the BMI groups.

The prevalence of physical inactivity was lower among individuals with a high education level across all BMI groups. The highest difference was found in the normal-weight individuals (22.3%) and the lowest difference was found for individuals with obesity grade III (14.9%). With an increase in age, the proportion of people not exercising was higher. The percentage of physical inactivity in the age group of 18–29 years was significantly higher among individuals with obesity grade III (44.2%) compared to normal weight individuals (17.5%). This difference disappeared in the age group of ≥ 80 years (normal weight: 59.5%; obesity grade III: 68.2%). The percentage of physical inactivity was lower among those with higher household income. Fig 2 demonstrates the percentage distribution of each variable across the BMI groups.

### Relationship between socio-demographic variables and the risk of physical inactivity

Table 3 presents the results of the logistic regression analyses examining the relationship between the socio-demographic variables and the risk of physical inactivity. Being male was found to be associated with a slightly lower risk of physical inactivity compared to females, significant results were revealed for overweight (OR: 0.90, 95% CI: 0.82, 0.98), obesity grade I (OR: 0.82, 95% CI: 0.71, 0.95) and grade II (OR: 0.75, 95% CI: 0.56, 0.99). Compared with the reference age group of 18–29 years, the odds of physical inactivity were higher in the age groups of 30–59 years and ≥60 years. The highest risks were found for adults with obesity grade I (OR_30-<60_: 2.04, 95% CI 1.48, 2.80; OR_60-<80_: 2.12, 95% CI 1.51, 2.97) and normal weight (OR_30-<60_: 2.04, 95% CI 1.84, 2.28; OR_60-<80_: 1.98, 95% CI 1.72, 2.28). In terms of household income, the odds for the top 20% group were between 7% (OR_obesityIII_: 0.93, 95% CI: 0.44, 1.99) and 44% (OR_overweight_: 0.56, 95% CI: 0.48, 0.65) lower than that for the bottom 20% group. The odds among people with a high education level were between 35% (OR_obesityII_: 0.65, 95% CI: 0.45, 0.94) and 58% (OR_normalweight_: 0.42, 95% CI: 0.38, 0.46) lower than in people with a low education level. Among ethnic minorities, the risk of inactivity was higher for two-sided migration (both parents belong to ethnic minorities) for normal weight, overweight and obesity grades I and III. For obesity grades I and II, higher odds were found for a one-sided migration (one parent belonging to an ethnic minority) compared to the reference group. Being unemployed was found to be associated with a significantly higher risk for normal weight (OR: 1.69, 95% CI: 1.44, 1.98) and overweight (OR: 1.48, 95% CI: 1.24, 1.76). Having diabetes was associated with a significantly higher risk of inactivity in normal weight (OR: 1.73, 95% CI: 1.34, 2.22), overweight (OR: 1.33, 95% CI: 1.11, 1.58) and obesity grade I (OR: 1.70, 95% CI: 1.38, 2.10).

**Table 3:** Binary logistic regression on dependent variable physical inactivity for the BMI sample^a^.

	18,5 -< 25 (kg/m^2^)	25 -< 30 (kg/m^2^)	30 -< 35 (kg/m^2^)	35 -< 40 (kg/m^2^)	≥ 40 (kg/m^2^)
Variable category	OR (95% CI)	OR (95% CI)	OR (95% CI)	OR (95% CI)	OR (95% CI)
Male	0.96 (0.89–1.04)	0.90 (0.82–0.98)*	0.82 (0.71–0.95)*	0.75 (0.56–0.99)*	0.67 (0.44–1.04)
18 -< 30 years (Ref.)					
30 -< 60 years	2.04 (1.84–2.28)	1.76 (1.50–2.07)**	2.04 (1.48–2.80)**	1.26 (0.69–2.29)	1.08 (0.52–2.28)
60 -< 80 years	1.98 (1.72–2.28)	1.76 (1.47–2.10)**	2.12 (1.51–2.97)**	1.52 (0.80–2.91)	1.08 (0.46–2.52)
Low education (Ref.)					
Middle education	0.56 (0.51–0.62)*	0.64 (0.58–0.71)**	0.69 (0.58–0.83)**	0.84 (0.60–0.97)*	0.81 (0.49–2.52)
High education	0.42 (0.38–0.46)	0.47 (0.42–0.52)**	0.51 (0.42–0.60)**	0.65 (0.45–0.94)*	0.57 (0.32–0.99)*
Income level bottom 20% (Ref.)					
Income level 20–40%	0.93 (0.83–1.05)	0.93 (0.81–1.07)	0.74 (0.59–0.92)*	0.64 (0.43–0.97)	1.06 (0.59–1.89)
Income level 40–60%	0.76 (0.67–0.85)	0.76 (0.67–0.87)**	0.76 (0.61–0.94)*	0.62 (0.41–0.95)*	0.84 (0.44–1.60)
Income level 60–80%	0.67 (0.59–0.77)	0.68 (0.59–0.79)**	0.70 (0.55–0.89)*	0.40 (0.25–0.63)*	0.96 (0.46–2.03)
Income level top 20%	0.57 (0.50–0.66)	0.56 (0.48–0.65)**	0.64 (0.50–0.83)*	0.70 (0.42–1.16)**	0.93 (0.44–1.99)
No migration (Ref.)					
One-sided migration	0.90 (0.74–1.10)	0.91 (0.73–1.13)	1.26 (0.91–1.74)	1.32 (0.73–2.39)	0.93 (0.34–2.50)
Two-sided migration	1.53 (1.34–1.74)*	1.13 (0.96–1.34)	1.03 (0.77–1.37)	0.85 (0.51–1.44)	1.39 (0.56–3.46)
Unemployed	1.69 (1.44–1.98)**	1.48 (1.24–1.76)**	1.17 (0.89–1.52)	1.11 (0.74–1.68)	1.05 (0.57–1.95)
Diabetes	1.73 (1.34–2.22)**	1.33 (1.11–1.58)*	1.70 (1.38–2.10)**	1.34 (0.96–1.87)	0.99 (0.60–1.63)

Note: OR > 1 indicating a higher risk of reporting not to engage in PA.

* = p-value <0.05,

** = p-value <0.001, OR = Odds Ratio, 95% CI = 95% confidence interval of the odds ratio.

^**a**^ Model adjusted for sex, age, education, household income, migration background, employment, diabetes.

## Discussion

### Summary of results

The current study aimed to examine patterns of physical inactivity (defined as not taking part in leisure-time PA) and to identify socio-demographic predictors of physical inactivity among different BMI groups and people with diabetes to support the development of targeted interventions promoting PA among individuals with obesity and diabetes. Overall, our results revealed that in obese as well as in individuals with diabetes, socio-demographic predictors of physical inactivity were similar compared to those among normal weight and non-diabetic adults: a higher risk of inactivity was found in people of older age and those with low levels of education or low household income. The prevalence of physical inactivity was higher among people with obesity and diabetes than among normal weight and non-diabetic individuals, respectively. Considerable differences were also found between the obesity grades, with an increase of 11.7% from grade I to grade III. Nevertheless, every eighth obese and third diabetic person reported being physically active during their leisure-time for more than two hours per week, which is in accordance with the German recommendations for physical activity [39].

### Implications for PA interventions

The calculated extent of PA corresponds to earlier findings from Germany [40] and other countries [20, 41] confirming the well-established relationship between physical inactivity and obesity. The lack of PA among people with obesity and diabetes is problematic given the importance of adequate PA for the treatment and control of both diseases [42]. Those affected may have additional barriers to PA such as more serious physical disabilities [43], perceived discomfort during training [44], and feeling inferior and inadequate [45, 46] which influence their willingness to be physically active. Nevertheless, some individuals with obesity and diabetes engaged in leisure-time PA, even for more than four hours weekly. This indicates that barriers to PA among this population can be overcome. In this regard, knowledge regarding the specific challenges obese and diabetic adults face when it comes to PA is important for planning interventions that target them [47].

In addition to disease-related barriers, this study demonstrates that socio-demographic factors may be fundamental in influencing the PA behaviour of adults who are obese or diabetic. The key role of socio-demographic factors was observed across all subgroups independent of body weight and the presence of diabetes. In accordance with previous studies, our results indicated a lower risk of physical inactivity among men [48, 49]. In the present study, markers of low socioeconomic status such as low educational levels and less household income were associated with a higher risk of physical inactivity, consistent with earlier studies [20, 50–52]. Individuals with lower socioeconomic status generally appear to have greater barriers to exercise [53]. This implies the need for policy makers to consider social gradients when designing PA programs particularly if they target diabetic or obese adults.

Our findings support previous research demonstrating obese and diabetic older people to be particularly physically inactive [54–57]. Given the increasing proportion of these groups in the general population, health care providers should actively approach affected older people for PA interventions motivating them to engage in regular PA. The high level of physical inactivity among the elderly population can result from physical limitations due to age-related loss of physical function [58] or specific motivation barriers [41, 59]. However, obese, and diabetic people face additional limitations. Diabetes is associated with a risk of mental disability, which may further lead to lower participation in PA [56]. In addition, the functional effects of obesity may exacerbate the age-related decline in physical functions, and it has been found that the self-reported mobility of obese people is reduced compared to lean older adults [60]. When developing PA interventions for older people, specific disease-related conditions of obesity and diabetes which influence PA behaviour should also be considered [56].

Earlier studies are consistent with our results, indicating a higher risk of physical inactivity among people with an migration background [18, 61, 62]. Migrant populations in high-income countries are particularly affected by type 2 diabetes and obesity, underlining the need for PA interventions for these groups [63]. When planning such interventions, the existence of migration-related barriers on multiple levels should also be considered, including cultural and religious beliefs, environmental barriers, and (inter-)personal factors [64–67]. Programmes to improve access to sports clubs for migrants, such as the programme ‘Integration through Sport’ of the German Olympic Sports Federation [68], are promising. However, there is a need for interventions that specifically target older migrants as well as those with obesity and diabetes to positively influence their PA behaviour.

### Strengths and limitations

We pooled data from several public-health surveys to create a large and nationwide sample. For countries such as Germany, this is the only means to yield comprehensive information on specific target groups with different socio-demographic and health-related backgrounds. We were therefore able to analyse the outcomes stratified by three grades of obesity, which has rarely been examined previously in this context [69]. The selected variables are robust and have withstood the test of time, which justifies the pooling of surveys from different years. Consideration must be given to potential limitations related to the pooling procedure. The cross-sectional design precludes the assessment of causal relationships. Moreover, the pooling and self-report assessment design prevented the gathering of precise information about PA behaviour (such as intensity and duration) or type of diabetes. The interpretation of the ‘no PA’ group as an indicator of physical inactivity must be assessed with caution. In several data sets people were coded as physically inactive, when they reported a lack of PA in the past week, while in others they were asked whether they were active in a typical week. Future studies should also include PA in other domains to provide a more complete picture of the ‘real’ overall levels of PA considering that these could also promote health. In particular, the assessment of PA in the domains of work or for transport would have been quite relevant in this regard. Self-reported data has further restrictions as the accuracy of self-reports is often limited due to recall errors, social desirability bias and misinterpretation of questions (i.e., differences in the interpretation of the term PA [70, 71]). Moreover, studies generally report a poor concordance between self-report and objective measures of PA [72]. However, the possibility of recall and measurement errors tend to be greater for light and moderate intensity activities than for vigorous ones [73]. High correlations between self-reported and measured height and weight have been reported [74]. Nevertheless, overweight and obese people tend to underestimate their body weight [75] and at the same time to overestimate their PA to an even higher degree than normal weight subjects [76]. In addition, although BMI is a typical method of measuring obesity, it is a poor diagnostic tool for identifying obesity in the general population [77].

## Conclusion

In conclusion, the current study confirms that adults in Germany with diabetes or obesity are less physically active at leisure-time than those without diabetes and normal BMI, respectively. Identical socio-demographic factors for engaging in PA appears to be crucial in all groups. This will require intervention approaches to increase PA that reach the identified vulnerable target groups, particularly the older population and adults with a migration background who are also affected by obesity or diabetes.

## Supporting information

S1 Fig Percentage distribution of PA (categories (frequencies) for the diabetes sample. (TIF)

S2 Fig Physical inactivity (in %) for education, household income and age across the diabetes groups. (TIF)

S1 Table Binary logistic regression on dependent variable physical inactivity for the diabetes sample. ^**a**^ Note: OR > 1 indicating a higher risk of reporting not to engage in PA. * = p-value <0.05, ** = p-value <0.001, OR = Odds Ratio, 95% CI = 95% confidence interval of the odds ratio.^**a**^ Model adjusted for sex, age, education, household income, migration background, employment, BMI.
(DOCX)

## Abbreviations

BMI: Body Mass Index
CI: Confidence Interval
OR: Odds Ratio
PA: Physical Activity
