# Supplementary figures and images for: Physical inactivity in healthy, obese, and diabetic adults in Germany: An analysis of related socio-demographic variables
Source: PLoS One. 2021 Feb 9;16(2):e0246634. doi: 10.1371/journal.pone.0246634 (PMC7872299; doi:10.1371/journal.pone.0246634)

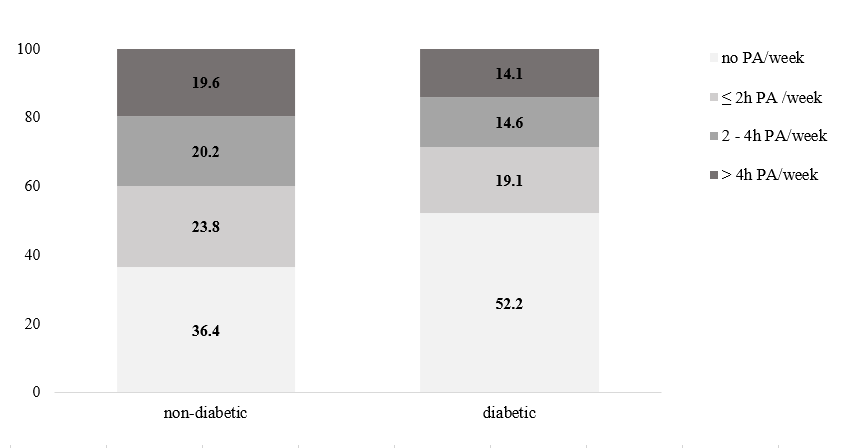

Supplement: S1 Fig — (TIF) [file pone.0246634.s001.tif]

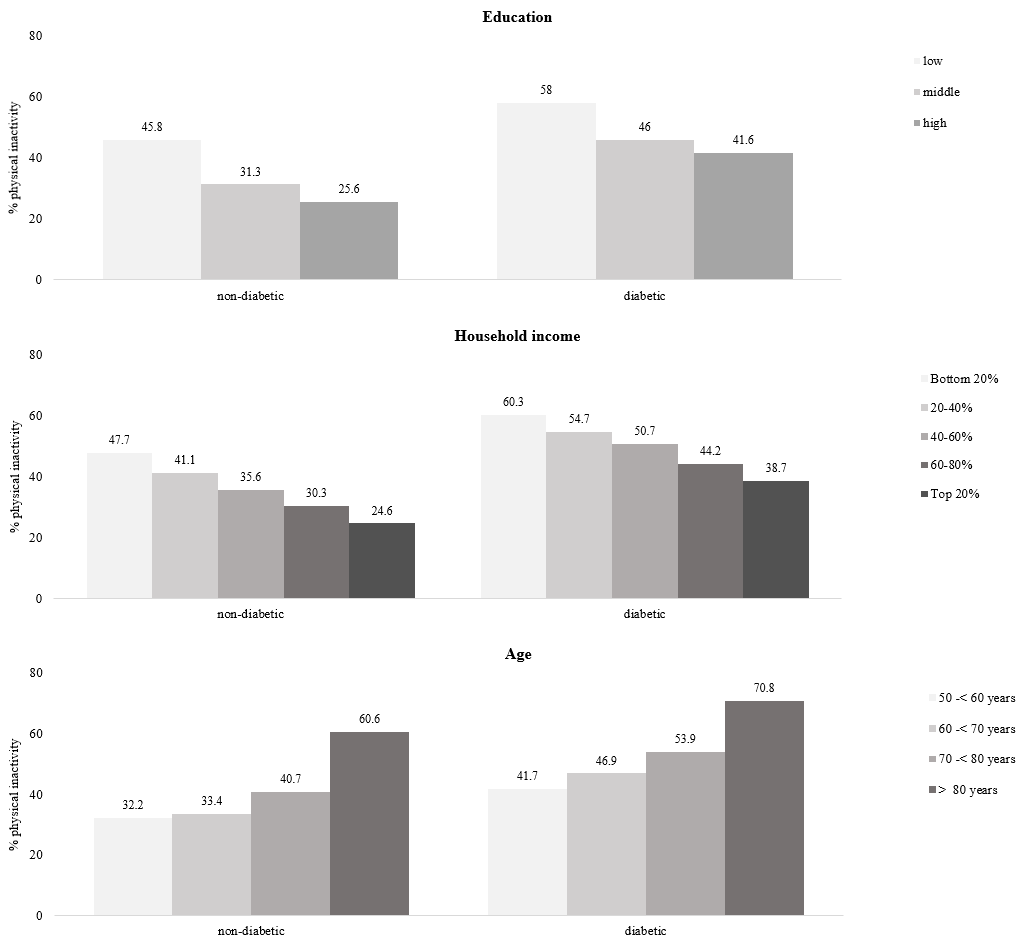

Supplement: S2 Fig — (TIF) [file pone.0246634.s002.tif]
