# Supplementary material for: Physical inactivity in healthy, obese, and diabetic adults in Germany: An analysis of related socio-demographic variables
Source: PLoS One. 2021 Feb 9;16(2):e0246634. doi: 10.1371/journal.pone.0246634 (PMC7872299; doi:10.1371/journal.pone.0246634)
Supplement: S1 Table — a Note: OR > 1 indicating a higher risk of reporting not to engage in PA. * = p-value <0.05, ** = p-value <0.001, OR = Odds Ratio, 95% CI = 95% confidence interval of the odds ratio.a Model adjusted for sex, age, education, household income, migration background, employment, BMI. (DOCX) [file pone.0246634.s003.docx]

**S1 Table. Binary logistic regression on dependent variable physical inactivity for the diabetes sample.**

|  | **Non-diabetic group** | **Diabetic group** |
| --- | --- | --- |
| **Variable category** | **OR (95% CI)** | **OR (95% CI)** |
| Male | 0.75 (0.69-0.82)** | 0.67 (0.53-0.85)* |
| 50 -< 60 years (Ref.) |  |  |
| 60 -< 70 years | 0.99 (0.92-1.07) | 0.95 (0.75-1.19) |
| Low education (Ref.) |  |  |
| Middle education | 0.67 (0.61-0.74)** | 0.63 (0.47-0.85)* |
| High education | 0.54 (0.49-0.60)** | 0.60 (0.44-0.80)* |
| Income level bottom 20% (Ref.) |  |  |
| Income level 20-40% | 0.82 (0.72-0.93)* | 1.17 (0.82-1.66) |
| Income level 40-60% | 0.68 (0.60-0.77)** | 0.92 (0.66-1.28) |
| Income level 60-80% | 0.60 (0.52-0.69)** | 0.78 (0.52-1.16) |
| Income level top 20% | 0.52 (0.46-0.60)** | 0.61 (0.40-0.92)* |
| No migration (Ref.) |  |  |
| One-sided migration | 0.99 (0.82-1.19) | 1.02 (0.59-1.76) |
| Two-sided migration | 1.11 (0.92-1.32) | 1.40 (0.82-2.37) |
| Unemployed | 1.40 (1-19-1.65)** | 1.34 (0.90-2.01) |
| Normal weight (Ref.) |  |  |
| Overweight | 1.15 (1.05-1.25)* | 0.79 (0.56-1.12) |
| Obesity grade I | 1.53 (1.36-1.72)** | 1.39 (0.97-1.98) |
| Obesity grade II | 1.79 (1.42-2.24)** | 1.48 (0.97-2.28) |
| Obesity grade III | 2.34 (1.61-3.38)** | 1.22 (0.70-2.12) |

**^a^** Note: OR > 1 indicating a higher risk of reporting not to engage in PA. *= p-value <0.05, **=p-value <0.001, OR = Odds Ratio, 95% CI = 95% confidence interval of the odds ratio.**^a^** Model adjusted for sex, age, education, household income, migration background, employment, BMI.
